# Solitary fibrous tumor within the mesorectum: literature review based on a case report of resection by transanal minimally invasive surgery (TAMIS)

**DOI:** 10.1007/s00384-024-04658-z

**Published:** 2024-06-07

**Authors:** Lennard Ströse, Moritz Sparn, Marie Klein, Luca Benigno, Stephan Bischofberger, Walter Brunner

**Affiliations:** 1 Department of General, Visceral, Endocrine and Transplant Surgery, Kantonsspital St. Gallen, 9007 St Gallen, Switzerland; 2 Department of Visceral Surgery, GZO Spital Wetzikon, 8620 Wetzikon, Switzerland; 3 Paracelsus Medical University, Salzburg, Austria

**Keywords:** Solitary fibrous tumor, Transanal surgery, Minimally invasive surgery, Pararectal tumor, TAMIS, SFT

## Abstract

**Purpose:**

Solitary fibrous tumors (SFT) are a rare entity of in majority benign neoplasms. Nevertheless, up to 20% of cases show a malignant tendency with local infiltration or metastasis. Commonly arising in the thoracic cavity, only few cases of SFT of the mesorectal tissue have been reported in the literature. Complete surgical resection, classically by posterior approach, is the treatment of choice. The purpose of this review is to demonstrate the safety and suitability of transanal minimally invasive surgery (TAMIS) as a surgical approach for the resection of benign pararectal solid tumors.

**Methods:**

We report the case of a 52-year-old man who was diagnosed incidentally with SFT of the distal mesorectum. Resection by TAMIS was performed. Based on this case, we describe the steps and potential benefits of this procedure and provide a comprehensive review of the literature.

**Results:**

Histopathology confirms the completely resected SFT. After uneventful postoperative course and discharge on day four, follow-up was recommended by a multidisciplinary board by clinical examination and MRI, which showed a well-healed scar and no recurrence up to 3 years after resection.

**Conclusion:**

SFT of the mesorectum is a very rare entity. To our knowledge, this is the first report on a TAMIS resection for SFT, demonstrated as a safe approach for complete resection of benign pararectal solid tumors.

## Introduction

A solitary fibrous tumor (SFT) is a rare variant of mesenchymal neoplasia. This entity was first described in the pleura by Klemperer and Rabin in 1931 [1, 2]. More than 50% of these tumors are located within the thoracic cavity, but also various sites, such as intraabdominal manifestations of the peritoneum, retroperitoneum and pelvis, liver, skin, head and neck or submandibular gland, have been described [3]. SFT in the pelvic region are a very rare entity with only a few similar cases reported in the literature. Surgical removal of tumors at this location can be challenging and is typically performed with invasive approaches, such as the Kraske procedure, while minimally invasive alternatives may offer potential benefits. We present a very rare case of an SFT in the mesorectal tissue that was resected by transanal minimally invasive surgery (TAMIS) and provide a comprehensive review of the literature.

## Methods and case presentation

**Fig. 1:**
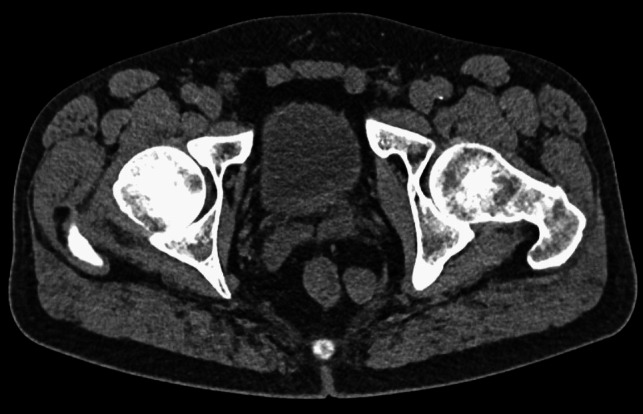
Pelvic CT scan showing a 2.3 × 1.9 × 2.0 cm soft tissue mass within the left mesorectal fascia

**Fig. 2:**
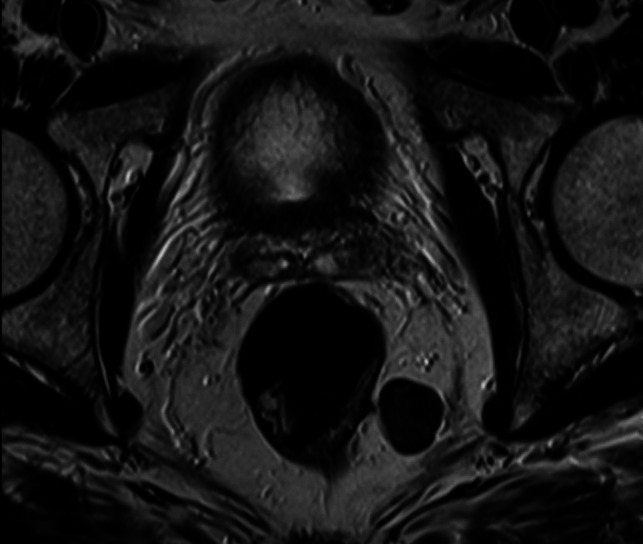
Pelvic MRI confirming the broad-based, noninfiltrative contact of the tumor with the levator ani muscle

A 52-year-old otherwise healthy caucasian male consulted the emergency department for left flank pain. After clinical examination, detection of microhematuria, and an inconclusive ultrasonography, abdominal CT scan confirmed an uncomplicated left-sided urolithiasis. Furthermore, an incidental finding of a 2.3 × 1.9 × 2.0 cm soft tissue mass within the mesorectal fascia on the left side adjacent to the pelvic diaphragm (Fig. 1) was revealed. No evidence of contact with or infiltration of the rectal wall, as well as no pathologically enlarged lymph nodes, was found. The proctological history was unremarkable with no local or systemic symptoms related to the tumor. Digital rectal examination revealed a deep, indolent, mobile mass located approximately 7 cm above the anal verge and 4 cm orally to the sphincter at the dorsolateral left side. Ten months before a colonoscopy with polypectomy of several benign adenomas was performed. Subsequent follow-up colonoscopy revealed no more abnormalities. Endosonography demonstrated a hypoechogenic, cystic, and indistinct mass in the perirectal tissues at the level of the seminal vesicles. Pelvic MRI confirmed the benign aspect of the mass and broad-based, noninfiltrative contact with the levator ani muscle (Fig. 2). Transanal biopsy of the tumor revealed cells of benign mesenchymal neoplasia. Thus, SFT was diagnosed. The case was discussed at the interdisciplinary tumor board for gastrointestinal neoplasia. Definitive treatment, and therefore resection of the tumor, was indicated due to the risk of malignant transformation and the absence of specific surveillance recommendations for SFT.

After discussion of the recommendation with the patient, we opted for a minimally invasive approach, including resection of the biopsy channel. We performed a transanal minimally invasive resection (TAMIS).

**Fig. 3:**
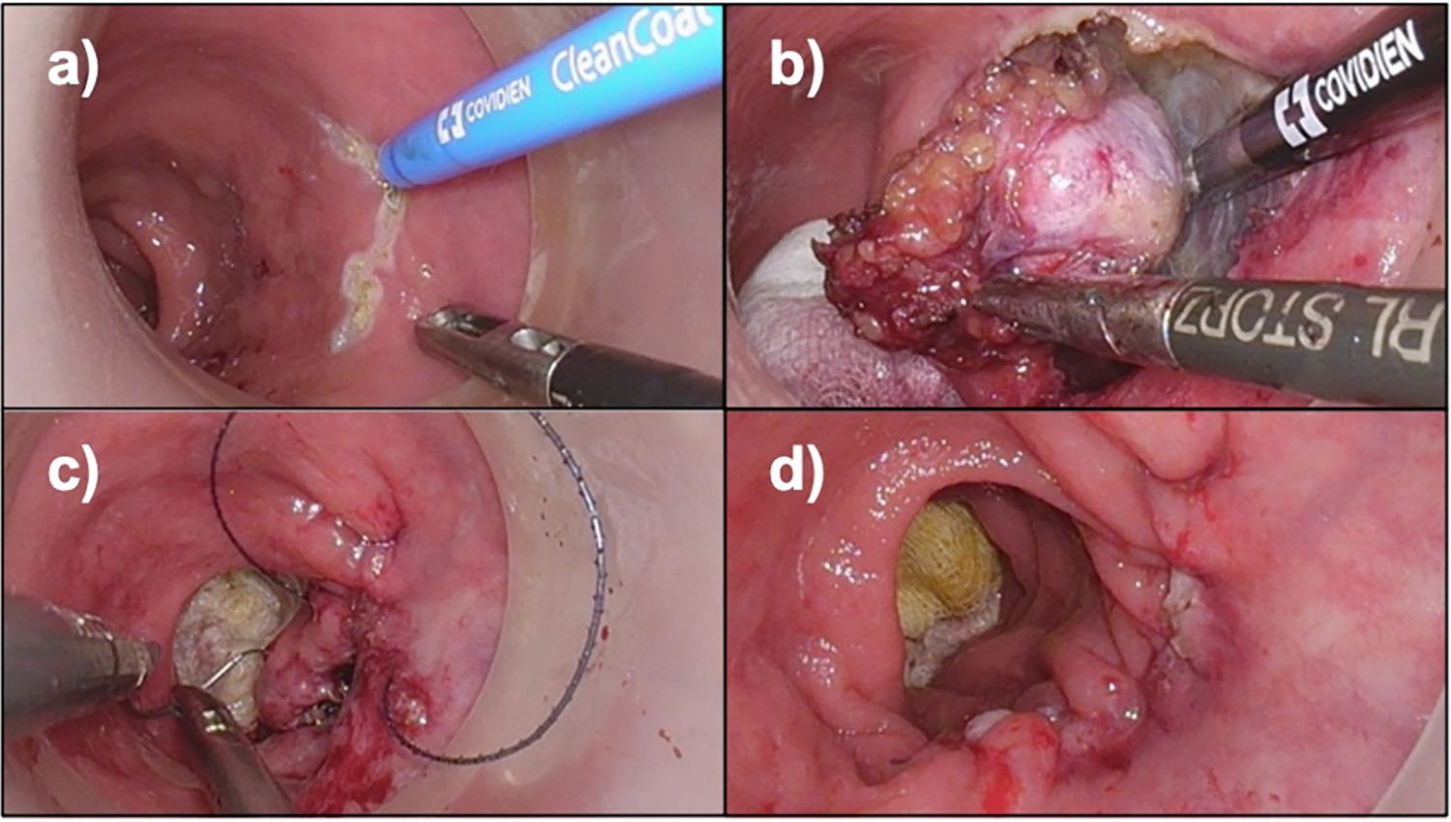
Intraoperative steps. **a** Incision of the rectal wall with diathermy hook. **b** En bloc dissection of the encapsulated tumor. **c** Performing a transanal running suture of the rectal wall incision. **d** Sutured rectal wall after resection

Operative procedure: After administering an enema, performing rectal irrigation, positioning of a GelPOINT Path transanal access platform (Applied Medical, California, USA) and insufflation with AirSeal (Conmed, New York, USA) up to 12 mmHg, a swab was placed orally to the palpable mass to preserve proper pneumorectum and vision. The tumor was clearly localized intraoperatively as a movable mass, palpable with both digital examination and laparoscopic instruments. The rectal wall was transmurally incised below the tumor with a diathermy hook (Fig. 3a), as favorably experienced in all our TAMIS and transanal total mesorectal excision (TME) cases. The encapsulated neoplasia was completely dissected in a no-touch technique with a safety margin of the rectal wall to ensure inclusion of the biopsy channel created during endosonography in a straight direction to the tumor (Fig. 3b). The mesorectum was dissected with LigaSure™ Maryland (Medtronic, Dublin, Ireland) to avoid bleeding. The dorsal portion of the tumor was easily removed from the pelvic fascia with preservation of the inferior hypogastric nerves. No bleeding was observed. Easy removal of the en bloc resected specimen through the GelPoint Path was followed by a second washout. Finally, the rectal wall was continuously sutured (Fig. 3c/d) with an absorbable barbed suture (V-Loc™, Medtronic, Dublin, Ireland) and the swab was removed.

## Results

The postoperative course was uneventful, and the patient was discharged on postoperative day four. Histologic examination of the resected tissue confirmed the diagnosis of SFT without any evidence of an aggressive clinical course. Due to the marginal resection margins (posterior side) and the risk of recurrence with possible malignancy, the multidisciplinary tumor conference decided on close follow-up (FU) with repeated clinical examinations and MRI. At the first postoperative FU at four weeks, the patient was in good health and asymptomatic. Rectoscopy showed a well-healed and nonirritating rectal scar. MRI control after 4 months and 3 years showed no evidence of recurrence. Three years after the resection, the patient still presented without symptoms or recurrence.

## Literature review and discussion

SFT are soft tissue tumors that can occur in diverse locations [3]. In addition to pleural manifestations, they have been described in the head and neck, including the meninges, larynx, and thyroid; intraabdominal, including the gastrointestinal and genitourinary tracts; parenchymal organs; and in soft tissues [3–13]. The abdominopelvic cavity has been described as a major primary site of SFT, accounting for up to 34% of cases, excluding meningeal manifestations [14]. Vallat-Decouceleare et al. suggest behavioral comparability between pleural and extrapleural SFT [15].

The incidence is similar in both sexes and the age at diagnosis is 50–60 years [3, 5, 14, 16]. The clinical presentation may be characterized by non-specific symptoms such as pain, hematochezia (e.g., in case of mucosal involvement) or compression-related signs due to the localization and extent of the tumor, such as urinary retention, bowel obstruction, or constipation [5, 8, 12, 16–18]. Paraneoplastic disease caused by SFT, e.g., Doege-Potter syndrome (hypoinsulinemic hypoglycemia due to overproduction of pro-IGF-2, a prohormone form of insulin-like growth factor 2), has also been described [19–21]. SFT can also be asymptomatic. Therefore, initial identification may occur as an incidental finding, as in our patient.

Diagnosis is based on radiological (CT/MRI) findings and histological examinations in addition to the clinical examination [6]. FDG-PET may be useful in differentiating between benign and malignant disease, but can be false-negative [19, 22, 23]. Due to its rarity, non-specific growth pattern and various localizations, the differential diagnosis is broad [24, 25]. Typical sensitive immunohistochemical markers that are highly expressed in up to 90% of SFT are CD34, BCL-2, and CD99 [26]. Strong expression of STAT6 is considered a sensitive and specific marker due to its association with the NAB2-STAT6 gene fusion [26–28].

Although the majority of SFT are benign, up to 20% of cases show a malignant tendency with local infiltration or metastasis [3, 6, 7]. For example, Schulz et al. described a low expression of CD34 and a high expression of IGF-2 to be significantly associated with malignant behavior [29]. Histologically malignant features may lead to worse outcomes with reduced overall survival [3, 7]. However, the value of percutaneous fine needle biopsy (FNB) is controversial due to the risk of tumor seeding [5, 7, 30].

In the present case, evidence of malignancy would have changed the therapeutic strategy. Transanal biopsy was possible due to the distal location of the tumor and the intended TAMIS provided the opportunity to resect the biopsy channel during tumor resection surgery. Therefore, we considered the risk of relevant tumor seeding to be negligible in this case and opted for a transmucosal biopsy as additional preoperative diagnostic tool.

Complete surgical resection with tumor-free resection margins is the treatment of choice [3, 5, 7, 16, 22, 24, 31–34]. The extent of resection depends, among other factors, on the risk of malignancy [31]. Because tumor-related symptoms may be present only in extensive disease, diagnosis may be delayed. Wang et al. reported diameters up to 28 cm and in 2015 Yokoyama et al. presented a case with a tumor measuring 30 cm in largest diameter [16, 35]. Complete resection at this stage can be challenging [35].

In the literature, a posterior approach (e.g., Kraske procedure) is classically described for the resection of retro- or pararectal tumors, especially in cases of tailgut cysts [5, 30, 36]. Alternatives are transabdominal, transvaginal, transrectal, or combined approaches [30, 31, 34, 37, 38]. Abdominoperineal resection or robotic-assisted resection has also been described [25, 39]. The indications for the different approaches may overlap depending on size, localization, and positional relationship or even infiltration of surrounding structures [31, 37]. Due to the potentially hypervascular nature of SFT, severe bleeding may occur during surgery [40]. Therefore, concepts for the pre- or periprocedural reduction of the blood supply to the tumor should be considered [41]. In suitable cases, embolization of feeding arteries may be useful to facilitate safe surgical resection or biopsy [5, 35]. Additionally, intraoperative temporary percutaneous balloon occlusion of the abdominal aorta has been reported for the resection of pelvic tumors and even for the resection of intrapelvic SFT [42, 43].

A beneficial effect of neoadjuvant or adjuvant radiotherapy has not yet been well evidenced but remains controversial [3, 16, 43–45]. However, in selected cases, primary or adjunctive radiotherapy and/or chemotherapy might still be considerable. Possible indications may include malignancy with infiltrative growth or disseminated metastases, postoperative positive margins with no possibility of resectability, or severe paraneoplastic syndromes [46–50]. De Boer et al. were able to demonstrated in a case of paraneoplastic active SFT that combined chemoradiotherapy with embolization can reduce tumor size and hormone activity to achieve resectability [19]. However, embolization is not considered a definitive treatment [41].

Overall and in accordance with literature, we consider an individualized, multidisciplinary approach for each patient mandatory.

In our presented case, we performed a transanal minimally invasive approach, which we considered most appropriate due to the distal pararectal side and size of the tumor between the mesorectum and the pelvic floor and its low-grade histologic and radiologic profile. Compared to the literature, our patient's tumor was small with a maximum diameter of 2.3 cm, so we expected to achieve adequate visualization and removal through the anal canal [16].

To our knowledge, this is the first description of a TAMIS resection of a pararectal SFT.

The known advantages of TAMIS include good and ergonomic visualization of the surgical field, the possibility of tension-free intraluminal closure of the rectal incision, reduced risk of iatrogenic sacral nerve injury and reduced postoperative pain, in part due to the absence of external wounds and skin incisions [51]. Thus, postoperative morbidity can be reduced and recovery is faster [51, 52]. Postoperative bleeding, urinary retention, pelvic abscess, or peritoneal penetration are the most common complications [52].

Several studies reviewed by Kim et al. in 2021 have proven TAMIS to be safe and effective for oncologic and postoperative outcomes [52, 53]. TAMIS also offers the option to remove the transmucosal biopsy channel, perform transanal reoperation in case of R1 resection or complication and it can be combined with other surgical techniques [52]. Compared to transanal endoscopic microsurgery (TEM) or robotic TAMIS (r-TAMIS), conventional TAMIS is economical and allows surgeons to use familiar laparoscopic instruments [52, 54–56]. For experienced laparoscopic surgeons, Lee et al. in 2018 described a learning curve of 14–24 cases to achieve an adequate level of competence in TAMIS for local resections of rectal neoplasms [57]. As shown in the presented case, TAMIS can also be a safe and suitable approach for local resection of pararectal neoplasms. It remains to be seen what long-term impact robotics will have on the development of TAMIS.

Thus far, the patient presented has had an individualized FU with clinical examinations for 3 years and MRI 4 months and 3 years after resection. Actually, there is no proper guideline for the FU of SFT. Vallat-Decouceleare et al. reported on 92 extrathoracic SFT cases identified in nine international centers. The overall local recurrence rate was 4.3%, and metastasis occurred in 5.4%. Ten patients with either recurrence or at least one atypical histologic feature at the time of diagnosis were followed up for 10–180 months. Of these, eight (80%) experienced local or distant relapse, with generally higher grade pathology compared to primary tumors [15]. Gold et al. reported a slightly but significantly increased local recurrence rate for extrathoracic SFTs compared to intrathoracic tumors, and further literature suggests an unfavorable prognosis for intraabdominal or retroperitoneal tumor site [33, 58, 59]. The disease-specific survival rate is reported to be 89% after 5 years and 73% after 10 years [14]. Due to the possibility of late recurrence with an often asymptomatic course, long-term surveillance up to 15 years or more with closer FU within the first 2 years postoperatively seems to be beneficial, depending on the individual malignant potential of the resected tumor [3, 5, 7, 22, 25, 33, 60, 61]. In 2020, Ros et al. suggested a malignancy-based FU according to the National Comprehensive Cancer Network guidelines for soft tissue sarcomas [3]. Parameters associated with worse outcome are listed in Table 1. For example, these appear to be advanced age, high mitotic and proliferation rates, large tumor size, necrosis, positive surgical margins, or molecular biological features like mutation or dysfunction of TERT gene, TP53 or APAF1 [14–16, 22, 29, 33, 58–60, 62–71]. Based on four of these parameters, Demicco et al. proposed a model for risk stratification into groups of low, intermediate, and high overall risk for metastasis and mortality [14, 65]. Using this risk assessment model, our patient’s tumor had a low risk of metastatic activity.

**Table 1:** Parameters associated with unfavorable prognosis

**Author (et al.)**	**Year**	**Mitotic index ≥ 4 / 10 HPF**	**Tumor size ≥ 10 cm**	**High tumor cellularity**	**Tumor necrosis or hemorrhage**	**Molecular biological features**	**Immuno-histological features**	**Clinical features**	**Histologic features**
Sugita et al. [63]	2022		x				Higher Ki-67 labeling index	Advanced patient age	
Bianchi et al. [69]	2020					TERT gene mutation			
Georgiesh et al. [64]	2020	x			x			Male sex	
Park et al. [70]	2019					TP53 dysfunction, APAF 1 dysfunction			
Yamada et al. [58]	2019		x^a^					Central nervous system location, hypoglycemia, male sex	Dedifferentiation
Olson and Linos [24]	2018								Dedifferentiation
Demicco et al. [14, 65]	2017 2012	x	x		x^b^			Advanced patient age	
Kim et al. [66]	2017	x	x	x	x				Nuclear pleomorphism
Salas et al. [60]	2017	x			x			Advanced patient age, viscera location, location other than limb, radiotherapy	
Bahrami et al. [71]	2016					TERT gene mutation			
Pasqualli et al. [67]	2016	x		x					Cellular atypia
Schulz et al. [29]	2014				x		Low expression of CD34, high expression of IGF2		
Cranshaw et al. [59]	2009	x		x	x			Retroperitoneal or intraabdominal localization	Cytological atypia, infiltrative margins
Gold et al. [33]	2002		x					Extrathoracic location, recurrent disease on presentation	Positive surgical margins, malign appearance
Vallat-Decouvelaere et al. [15]	1998	x		x	x				Nuclear atypia
England et al. [68]	1989	x	xª	x	x				Nuclear pleomorphism

^a^Tumor size ≥ 5 cm

^b^Significant for metastatic disease, not significant for mortality

## Conclusion

SFT of the mesorectum is a very rare entity. To our knowledge, we are the first to report the resection of a mesorectal SFT performed by TAMIS. The presented case confirms that in selected patients, TAMIS is suitable and safe for resection of distal pararectal solid tumors. Experience with transanal minimally invasive surgery is mandatory. Due to the rarity and variability of the disease, there are mainly case reports and case series available and studies with larger patient numbers are difficult to achieve. A better comparability of the different approaches, corresponding indications, and FU recommendations will therefore probably not be achieved, and individually tailored concepts will remain necessary.

## Data Availability

The authors confirm that the data supporting the findings of this study are precisely represented within the article. The data are not publicly available due to their containing information that could compromise the privacy of the research participant. Further anonymized data are available on request from the corresponding author.
